# Limited Nerve Regeneration across Acellular Nerve Allografts (ANAs) Coincides with Changes in Blood Vessel Morphology and the Development of a Pro-Inflammatory Microenvironment

**DOI:** 10.3390/ijms25126413

**Published:** 2024-06-11

**Authors:** Jesús A. Acevedo Cintrón, Daniel A. Hunter, Lauren Schellhardt, Deng Pan, Susan E. Mackinnon, Matthew D. Wood

**Affiliations:** Division of Plastic and Reconstructive Surgery, Department of Surgery, Washington University School of Medicine, St. Louis, MO 63110, USA; jacevedo@wustl.edu (J.A.A.C.); hunterd@wustl.edu (D.A.H.); lmschellhardt@wustl.edu (L.S.); pandeng@wustl.edu (D.P.); mackinnons@wustl.edu (S.E.M.)

**Keywords:** allograft, nerve graft, nerve injury, nerve regeneration, peripheral nerve

## Abstract

The use of acellular nerve allografts (ANAs) to reconstruct long nerve gaps (>3 cm) is associated with limited axon regeneration. To understand why ANA length might limit regeneration, we focused on identifying differences in the regenerative and vascular microenvironment that develop within ANAs based on their length. A rat sciatic nerve gap model was repaired with either short (2 cm) or long (4 cm) ANAs, and histomorphometry was used to measure myelinated axon regeneration and blood vessel morphology at various timepoints (2-, 4- and 8-weeks). Both groups demonstrated robust axonal regeneration within the proximal graft region, which continued across the mid-distal graft of short ANAs as time progressed. By 8 weeks, long ANAs had limited regeneration across the ANA and into the distal nerve (98 vs. 7583 axons in short ANAs). Interestingly, blood vessels within the mid-distal graft of long ANAs underwent morphological changes characteristic of an inflammatory pathology by 8 weeks post surgery. Gene expression analysis revealed an increased expression of pro-inflammatory cytokines within the mid-distal graft region of long vs. short ANAs, which coincided with pathological changes in blood vessels. Our data show evidence of limited axonal regeneration and the development of a pro-inflammatory environment within long ANAs.

## 1. Introduction

Peripheral nerve injuries (PNIs) account for approximately 3% of trauma cases, which translates into more than 300,000 PNI cases annually in the United States [1,2,3]. The severity of PNI is classified based on the components of the nerve that are affected, where the most severe type of nerve injury is a complete transection of the nerve, resulting in a defect or gap between nerve ends. This type of nerve injury requires surgical intervention to bridge the nerve gap and facilitate axonal regeneration [4]. Autografts are considered the “gold standard” for surgically repairing nerve gaps but fail with increasing length and diameter of the graft. The harvest of autografts is technically challenging and time-consuming [5,6,7]. Acellular nerve allografts (ANAs) have been proposed as an alternative to replace autografts for all nerve gap injuries [8]. ANAs consist of chemically processed nerves that retain the extracellular matrix (ECM) and connective tissue layers of the nerve while lacking cells and major immunogenic components that could cause rejection of the transplanted allograft [2,7,9]. When repairing a nerve gap using an ANA, host cells will repopulate the ANA and promote the formation of a pro-regenerative environment for axonal regeneration [5,7,10]. When ANAs are used to repair short gaps (<3 cm), outcomes are comparable to autograft. However, ANAs used to repair long gaps (>3 cm) have been shown to result in suboptimal outcomes compared to their use in short gap repair [5,6,9]. The reasons for these outcomes are still being investigated.

Animal models of nerve gap repair using ANAs have recapitulated results and relationships observed in the clinic [11,12,13,14,15,16,17]. Specifically, as ANA length increases to accommodate the repair of long nerve gaps, this leads to limited axon regeneration across this longer ANA. To understand these limitations, studies have focused on identifying early differences (before 4 weeks post surgery) in the cell repopulation within ANAs based on graft length [10,12,13,14]. In previous studies, Schwann cells (SCs) within long ANAs developed a senescent-like phenotype with the limited expression of pro-regenerative factors in comparison to SCs within short ANAs [12,13]. Furthermore, these studies demonstrated that nerve repair using a short (2 cm) graft derived from a repopulated long (4 cm) ANA resulted in limited axon regeneration across this graft, suggesting that the failure in regeneration across long ANAs is due to the microenvironment that develops within the ANA rather than the inability of axons to grow across long distances [12,13,14].

More recent studies considering this same topic have revealed the role of the immune system [10,13,14,18,19,20,21]. Immune cells, such as macrophages, are among the first cells to be recruited to an injury site and repopulate acellular scaffolds, where they play a critical role in facilitating regeneration [7,22,23,24]. Infiltrating macrophages will react to the hypoxic environment within acellular scaffolds by secreting pro-angiogenic factors, such as vascular endothelial growth factor (VEGF), to promote angiogenesis. These newly formed blood vessels will promote SC migration and axonal growth into the scaffold [25]. Along those lines, previous studies have demonstrated that disrupting macrophage infiltration and angiogenesis within ANAs delays and interrupts regeneration across these ANAs [10]. Therefore, it is possible but unclear if differences from the immune response coordinating angiogenesis could lead to differences in blood vessels for ANAs based on the graft length.

In addition to orchestrating angiogenesis, immune cells can also alter the inflammatory microenvironment after nerve injury [14,24,26,27]. In a study comparing nerve regeneration after a crush injury between young and old mice, older mice had a delay in the activation of the inflammatory response after injury. This early delay in inflammation caused the development of a pro-inflammatory environment late in the regenerative process, which correlated with limited nerve regeneration [28]. Bringing this back to rodent models of nerve gap repair using ANAs, the early stages of nerve regeneration within ANAs have demonstrated differential expression of pro- and anti-inflammatory cytokines based on ANA graft length [14]. It is unknown, however, how the inflammatory response within short versus long ANAs changes as nerve regeneration progresses.

As previous studies have focused on understanding early events within the developing microenvironment of ANAs based on graft length, we instead chose to focus on characterizing the microenvironment of ANAs as regeneration further progressed. In this study, we used histology to obtain a visual description of the microenvironment within short and long ANAs at the early stages of nerve regeneration (before 4 weeks post surgery) as well as beyond (from 4 to 8 weeks post surgery). We also identified the time course of axonal regeneration and blood vessel formation and morphology between short and long ANAs. Finally, we measured broad inflammatory gene changes from the microenvironment within short versus long ANAs over this extended time course of regeneration.

## 2. Results

### 2.1. Axons Robustly Regenerate into the Proximal Graft of ANAs, Regardless of Length, but Long ANAs Contain Limited Axon Regeneration beyond This Region

#### 2.1.1. Minimal Differences in the Number of Myelinated Axons between the Proximal Grafts of Short and Long ANAs

**Figure 1:**
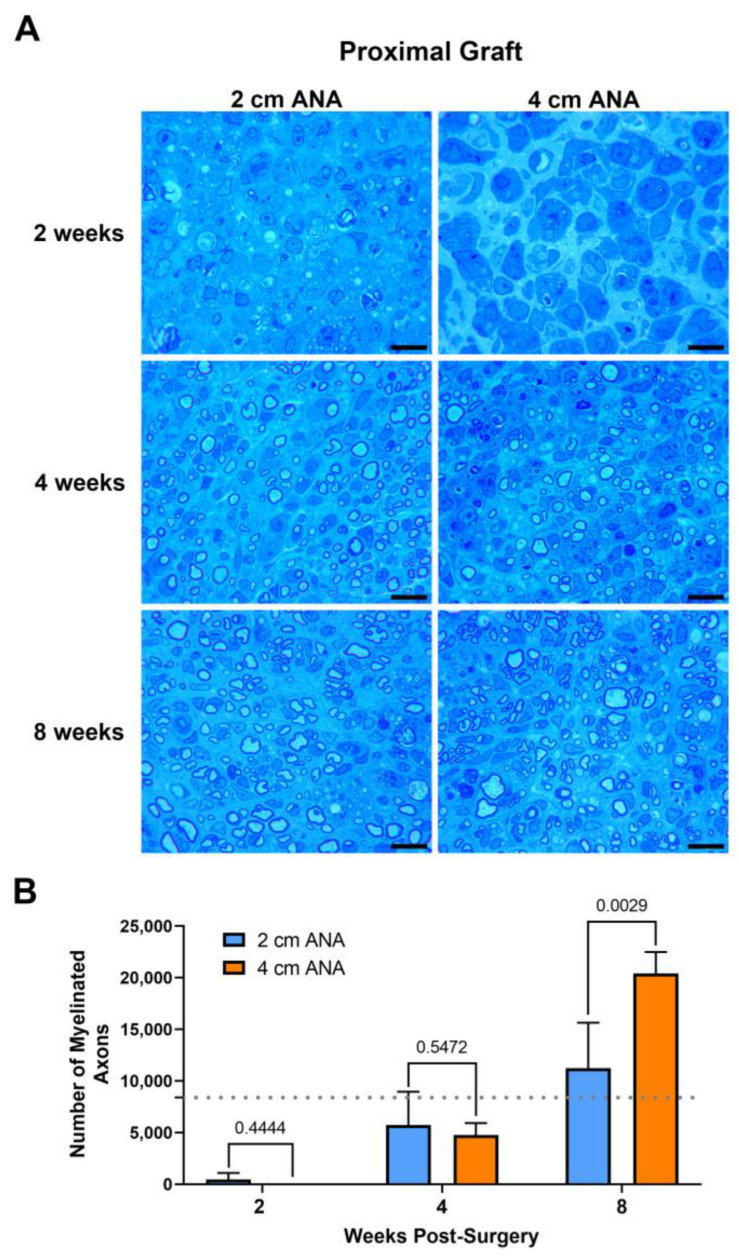
The number of regenerating myelinated axons within long (4 cm) ANAs surpasses short (2 cm) ANAs at the proximal graft region. Representative histological images of graft cross-sections (**A**) and quantification of the number of myelinated axons (**B**). Black scale bar is 10 µm. Data represented as mean ± SD (n = 5/group). *p* values are represented above each comparison. Dotted gray line represents average for uninjured sciatic nerve.

The repair of nerve gaps using either short (2 cm) or long (4 cm) ANAs revealed a relationship of increasing myelinated axon regeneration into either length ANA as time progressed. Assessment of myelinated axon counts within the proximal grafts of either ANA was not different by 2 or 4 weeks post surgery (Figure 1). By 8 weeks, the proximal graft of the long ANA contained a twofold greater number of myelinated axons than the short ANA. This number of myelinated axons contained in the proximal graft of either ANA surpassed the values for the uninjured sciatic nerve. However, also by 8 weeks, for the long compared to short ANAs, the area of the graft occupied by myelinated axons decreased by ~20%, and the size of myelinated axons decreased by ~50%, which was reflected in decreased myelination (Figure S1). Along with these changes, there was no difference for either ANA length in axonal and myelin debris or cross-sectional area of the graft across any endpoint. These results demonstrated that the proximal graft region of ANAs, independent of ANA length, supports axonal regeneration.

#### 2.1.2. Limited Regeneration across the Mid-Distal Graft of Long ANAs versus Short ANAs

**Figure 2:**
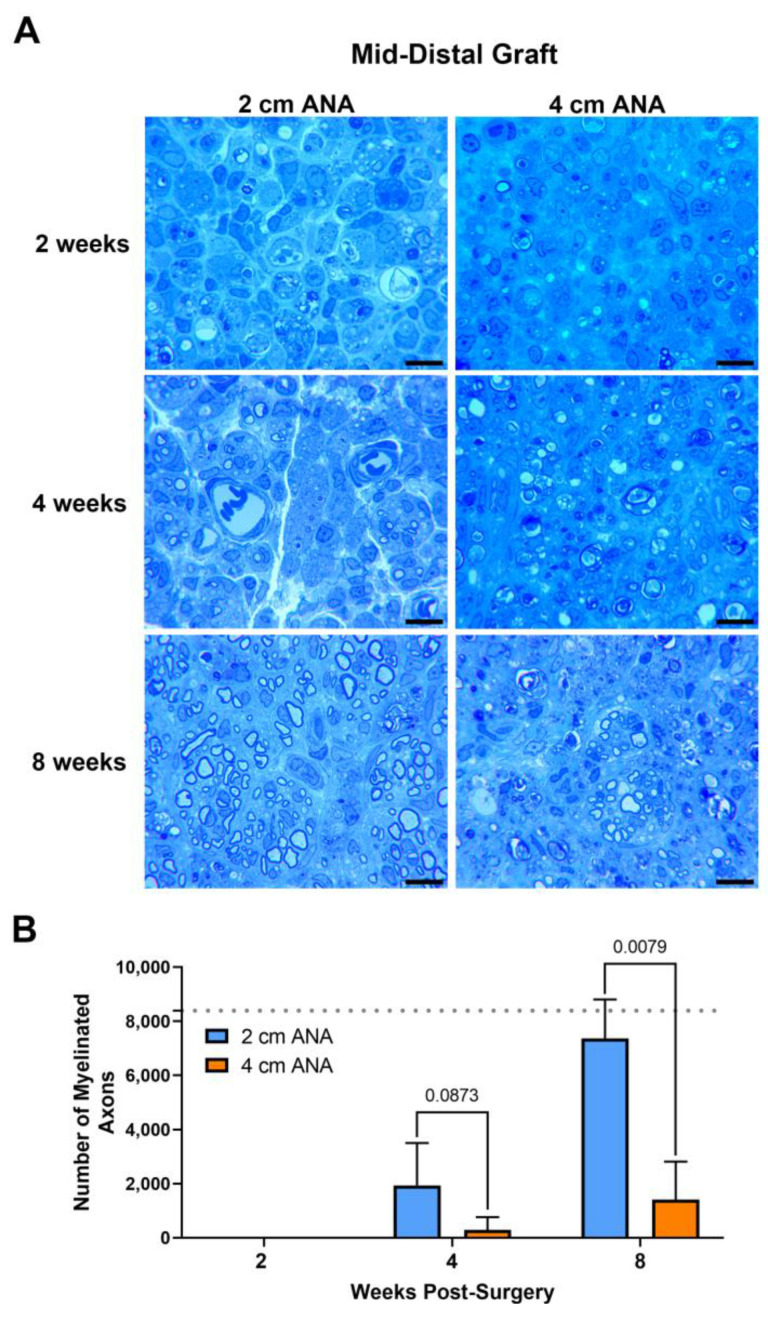
The number of regenerating myelinated axons within long (4 cm) ANAs is reduced when compared to short (2 cm) ANAs at the mid-distal graft region. Representative histological images of graft cross-sections (**A**) and quantification of the number of myelinated axons (**B**). Black scale bar is 10 µm. Data represented as mean ± SD (n = 5/group). *p* values are represented above each comparison. Dotted gray line represents average for uninjured sciatic nerve.

Assessment of myelinated axon counts within the mid-distal graft of long ANAs revealed a sixfold decrease in the number of myelinated axons in comparison to the short ANAs by 4 weeks post surgery. Additionally, at this endpoint, the size of myelin was reduced by ~20% in the myelinated axons within long ANAs. By 8 weeks, the mid-distal graft of long ANAs underwent a fivefold decrease in the number of myelinated axons when compared to the short ANAs (Figure 2). Similarly, the area of the graft occupied by myelinated axons in the mid-distal graft of long ANAs was reduced by ~80% in comparison to short ANAs (Figure S2). Interestingly, from the 4- to the 8-week endpoint, we observed a fourfold increase in the number of myelinated axons within the mid-distal graft, relative to their starting quantity, for either ANA (Figure 2). Further analysis of the mid-distal graft of long ANAs at 4 weeks post-surgery revealed a twofold increase in the amount of myelin and axonal debris when compared to the short ANAs. By 8 weeks, this twofold increase in long ANAs is maintained. However, the amount of debris from 4 to 8 weeks post-surgery is reduced by 50% in both short and long ANAs (Figure S2). Further analysis of the mid-distal grafts revealed no differences in the size of myelinated axons and the cross-sectional area of the grafts between the short and long ANAs. These results demonstrated the mid-distal graft of short ANAs supports robust axonal regeneration, while regeneration within the mid-distal graft of long ANAs is restrained.

#### 2.1.3. Fewer Number of Myelinated Axons Regenerated across Long ANAs into the Distal Nerve When Compared to Short ANAs

**Figure 3:**
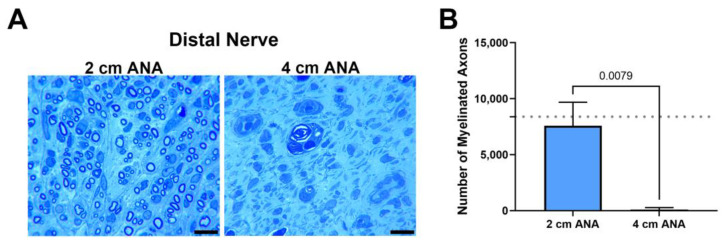
The number of regenerating myelinated axons reaching the distal nerve across long (4 cm) ANAs is inferior to short (2 cm) ANAs. Representative histological images of distal nerve cross-sections (**A**) and quantification of the number of myelinated axons (**B**). Black scale bar is 10 µm. Data represented as mean ± SD (n = 5/group). *p* values are represented above each comparison. Dotted gray line represents average for uninjured sciatic nerve.

Finally, as a hallmark of regenerative success, the number of myelinated axons contained within the distal nerve was quantified at 8 weeks, as 2 and 4 weeks was not sufficient time for axons to cross either ANA. The number of myelinated axons within the distal was markedly decreased for the long ANAs (98) in comparison with short ANAs (7583) (Figure 3). Taken together, these results demonstrated limited regeneration across long ANAs and successful regeneration across short ANAs.

### 2.2. Schwann Cell Quantities within the Mid-Distal Graft of Long ANAs Is Markedly Reduced When Compared to Short ANAs

**Figure 4:**
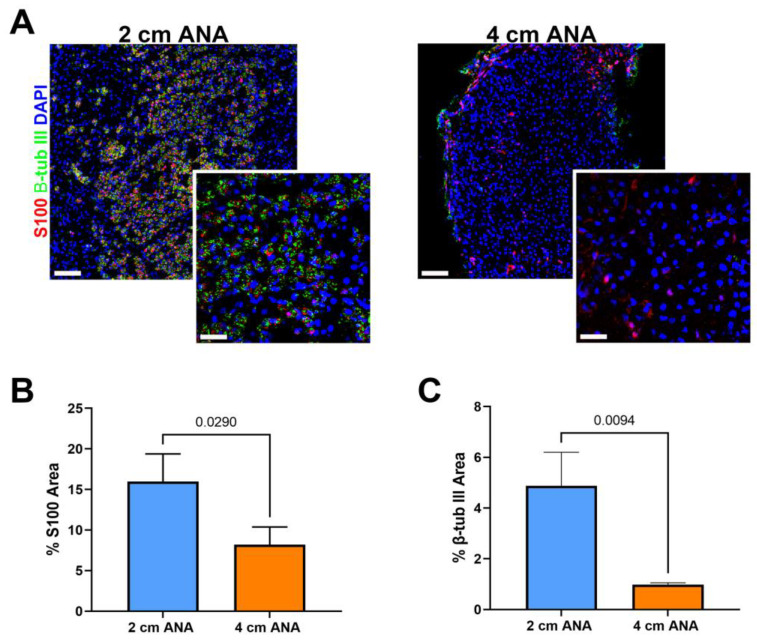
Expression of Schwann cells (SCs) and axon markers is reduced in long (4 cm) ANAs versus short (2 cm) ANAs at the mid-distal graft region. (**A**) Immunofluorescence images of graft cross-sections at 8 weeks post-surgery showing SCs (S100; red) and axons (β-III tubulin; green). Quantification of graft cross-sections for percent area stained (**B**) S100 and (**C**) β-III tubulin positive. White scale bar is 30 µm (20× magnification) and 10 µm (60× magnification), respectively. Data represented as mean ± SD (n = 3/group). *p* values are represented above each comparison.

Given the severely limited myelinated axon regeneration across long ANAs by 8 weeks post surgery, we further evaluated ANAs at 8 weeks using immunohistochemistry to evaluate the presence of any (myelinated or unmyelinated) axon regeneration (β-III Tubulin+) and Schwann cells (S100+). Interestingly, there was a 5% increase in S100+ area within the proximal graft of long vs. short ANAs, but no difference in β-III Tubulin+ area (Figure S3). Within the mid-distal graft of long ANAs, there was a twofold decrease in S100+ area and a tenfold decrease in β-III Tubulin+ in comparison to short ANAs (Figure 4). These results demonstrated overall decreased axon regeneration within the long ANA mid-distal graft region but also suggested fewer SCs within this region.

### 2.3. Progressive Changes in Blood Vessel Morphology within the Mid-Distal Graft of Long ANAs Significantly Differs from Short ANAs

#### 2.3.1. Histological Analysis shows Changes in Blood Vessel Morphology in the Mid-Distal Graft of Long ANAs

**Figure 5:**
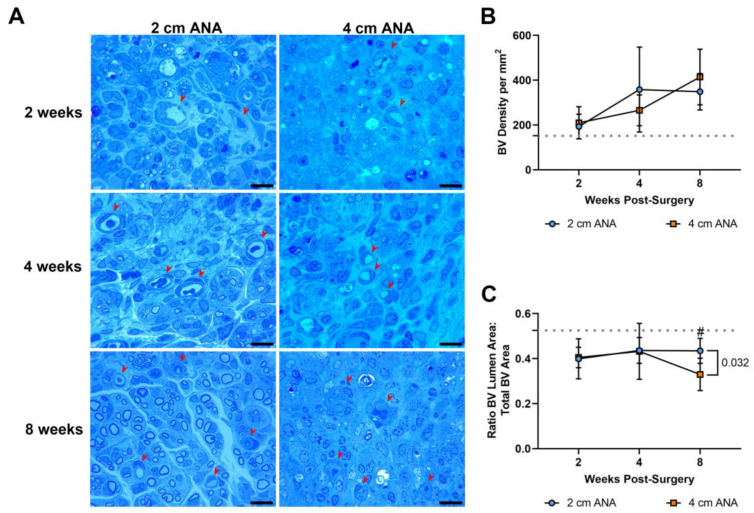
BV density does not differ based on ANA length, but BV morphology ultimately changes within the mid-distal graft of long (4 cm) ANAs. (**A**) Representative histological images of graft cross-sections showing BVs (red arrowheads) post-surgery. Quantification of (**B**) BV density and (**C**) BV morphology. Black scale bar is 10 µm. Data represented as mean ± SD (n = 5/group). *p* value is represented next to statistically significant comparisons. Dotted gray line represents the average for uninjured sciatic nerve.

As angiogenesis has a key role in nerve regeneration, the density and morphology of blood vessels were assessed in the previous histological sections over time. There was no difference in the density and morphology of the blood vessels in the proximal graft of either ANA (Figure S4). Similarly, there was no difference in the density of blood vessels between the mid-distal graft regions of either ANA (Figure 5). This was further confirmed with immunofluorescence showing no difference in the expression of endothelial cell marker RECA-1^+^ between short and long ANAs at 8 weeks (Figures S5 and S6). However, within the mid-distal graft regions of ANAs, the morphology of the blood vessels showed stark morphological changes that developed over time. While not different at 2 or 4 weeks post surgery, the mid-distal graft of long ANAs underwent a ~25% decrease in the lumen to BV area by 8 weeks compared to the short ANAs (Figure 5).

#### 2.3.2. Electron Microscopy Reveals Decrease Lumen Size and Thickening of Blood Vessel Walls in the Mid-Distal Graft of the Long ANAs Versus Short ANAs

**Figure 6:**
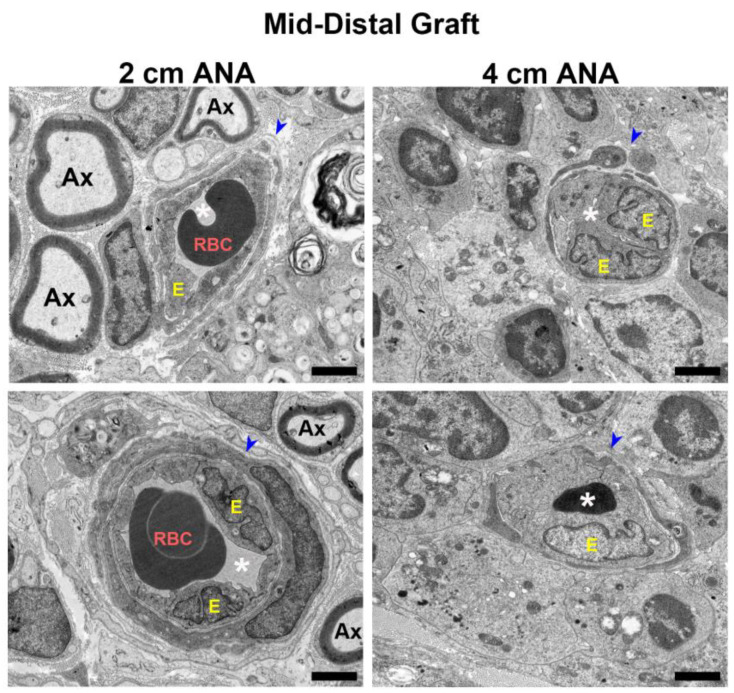
BVs within long (4 cm) ANAs have swollen endothelial cells and narrowed lumen. Representative images from electron microscopy of graft cross-sections. Notice endothelial cell swelling and decrease in lumen area in BV present in the mid-distal graft of the long (4 cm) versus short (2 cm) ANAs at 8 weeks post-surgery. Blue arrowhead indicates BVs; RBC indicates red blood cells; Ax indicates myelinated axons; white asterisk (*) denotes BV lumen; E indicates endothelial cells. Black scale bar is 2 µm.

To further examine the changes in blood vessel morphology, electron microscopy was used to reveal microstructural changes in the blood vessels at 8 weeks. Micrographs of the proximal grafts of either ANA showed blood vessels with unobstructed lumens and healthy blood vessel walls surrounded by myelinated axons (Figure S7). Conversely, when comparing the morphology of the blood vessels within the mid-distal graft of ANAs, the short ANAs contained blood vessels mirroring the proximal graft aspects just described, while the long ANAs had decreased and sometimes entirely obstructed lumens, as well as endothelial cell swelling and increased nuclei (Figure 6). Taken together, these results suggested blood vessels within the mid-distal graft of long ANAs underwent pathological changes as time progressed.

### 2.4. The Mid-Distal Graft within Long ANAs Develops into a Pro-Inflammatory Environment Relative to Short ANAs

#### 2.4.1. Increase Expression of Pro-Inflammatory Cytokine in the Mid-Distal Graft of Long versus Short ANAs

**Figure 7:**
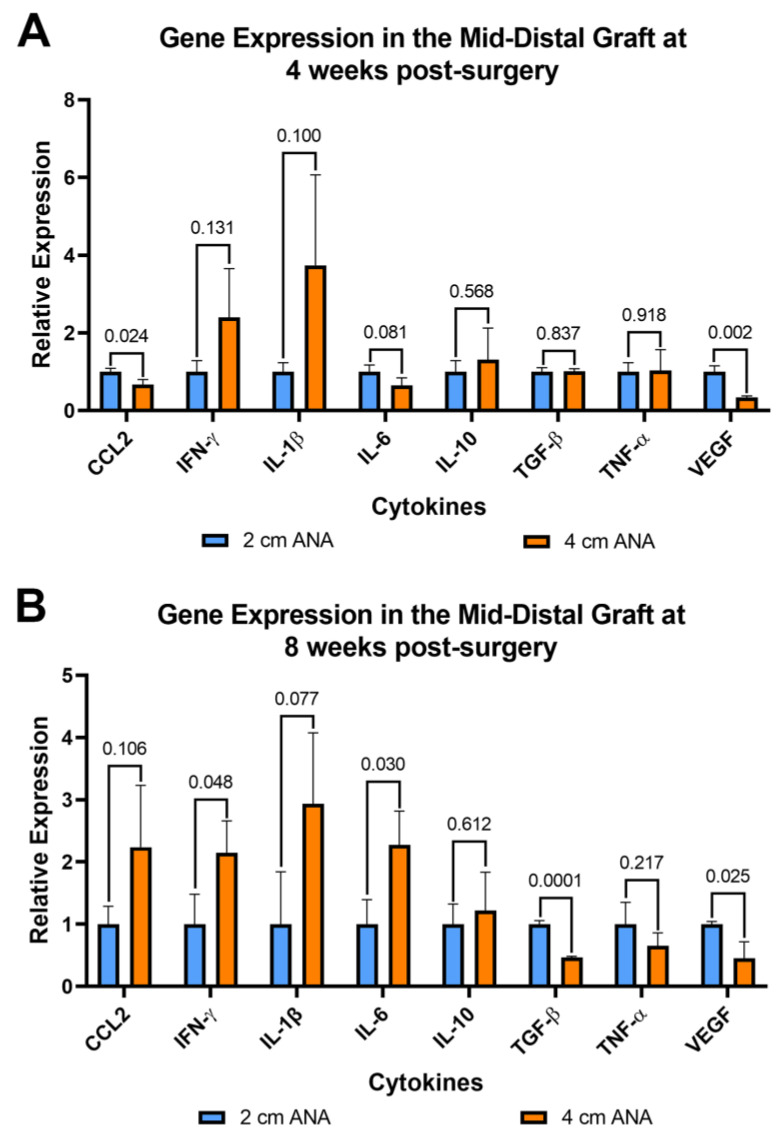
Gene expression of cytokines within long (4 cm) ANAs ultimately shows an increase in a pro-inflammatory state relative to the short (2 cm) ANAs. Expression levels are relative to 2 cm ANA measured at (**A**) 4 weeks and (**B**) 8 weeks post-surgery. Data represented as mean ± SD (n = 3/group). *p* values are represented above each comparison.

Since inflammation can affect both nerve regeneration and blood vessels, the expression of pro- and anti-inflammatory genes was evaluated for cells contained within ANAs. From cells within the proximal grafts of ANAs, the short and long ANA comparison generally did not differ in inflammatory gene expression levels, with some exceptions. At 4 weeks post surgery the expression of Vegf was decreased twofold in the long ANAs when compared to short ANAs. By 8 weeks, the long ANAs underwent a ninefold increase in Il-1b,a fourfold increase in Il-10, and a twofold decrease in Tgf-β in comparison to short ANAs (Figure S8). For the mid-distal graft comparison at 4 weeks post surgery, the long ANAs experienced a twofold and fourfold decrease in Ccl2 and Vegf, respectively, relative to the short ANAs (Figure 7A). By 8 weeks post surgery, the long ANAs had undergone a twofold increase in Il-6 and Ifn-γ and a twofold decrease in the expression of Tgf-β and Vegf in comparison to short ANAs (Figure 7B).

#### 2.4.2. Expression of Pro-Inflammatory Cytokines Increase in the Mid-Distal Graft of the 4 cm ANAs as Time Progresses

**Figure 8:**
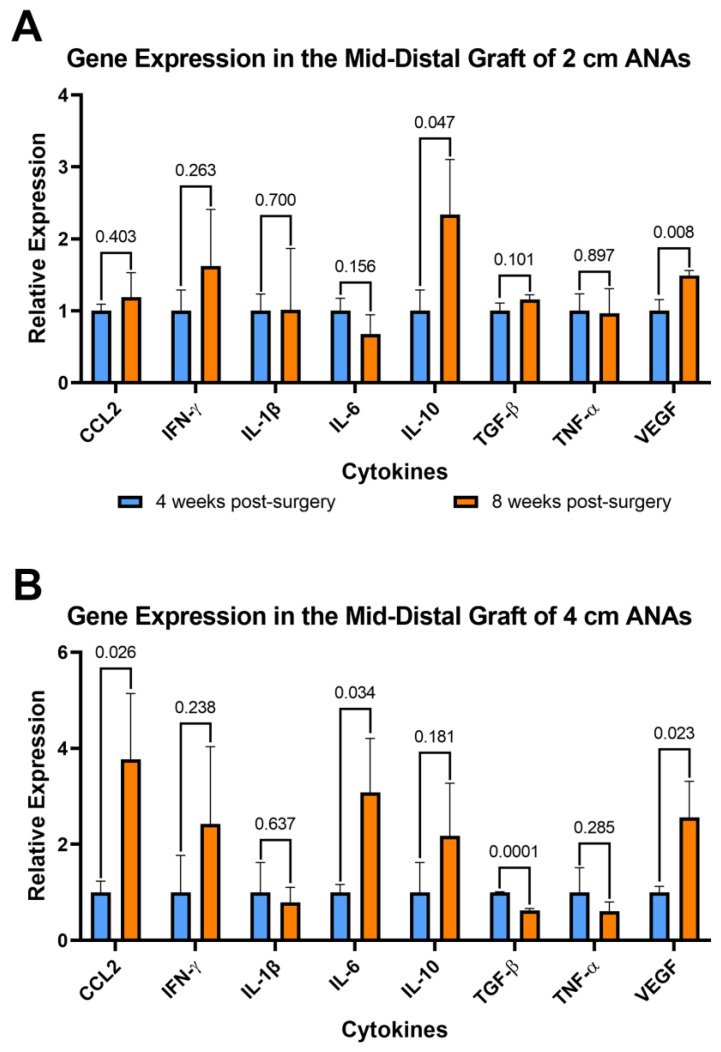
Gene expression of cytokines within long (4 cm) ANAs shows the development of a pro-inflammatory environment over time. Expression levels of 8-week data are relative to 4-week data measured for (**A**) short (2 cm) ANA and (**B**) long (4 cm) ANA. Data represented as mean ± SD (n = 3/group). *p* values are represented above each comparison.

To further assess the changes in inflammation over time, the expression of these genes was compared between the 4- and 8-week endpoints. Comparison within the proximal graft of short ANAs at these endpoints showed fivefold and twofold decreases in Il-1b and Il-6, respectively, at 8 weeks when compared to 4 weeks (Figure S9A). For comparison of the proximal grafts within long ANAs, we observed a twofold increase in Il-10 from 4 to 8 weeks (Figure S9B). Similarly, assessment of the mid-distal graft within short ANAs revealed a 150% increase in Il-10 and 50% increase in Vegf at the 8-week endpoint relative to 4 weeks (Figure 8A). Lastly, a comparison of the mid-distal graft within long ANAs showed a threefold increase in Ccl2 and Il-6, a twofold increase in Vegf, and a twofold decrease in Tgf-β as time progressed from 4 to 8 weeks (Figure 8B). Interestingly, we identified a trend in the proximal graft of long ANAs where we observed a threefold increase in Ccl2 and a twofold increase in Ifn-γ and Il-6 from the 4- to 8-week endpoints. Similarly, we observed a twofold increase in Ifn-γ in the mid-distal graft of the long ANA as time progressed. These results suggested that cells within short ANAs decrease the expression of pro-inflammatory genes as time progresses, while cells within long ANAs promote the development of a pro-inflammatory microenvironment. To further assess if the increased expression of pro-inflammatory cytokines is caused by a higher number of specific immune cells in the long ANAs, the number of macrophages between short and long ANAs was compared at 8 weeks. Immunofluorescence showed no difference in the number of Iba1^+^ macrophages between the short and long ANAs (Figures S5 and S6), suggesting the expression of a pro-inflammatory environment is not caused by an increased number of macrophages in the long ANAs.

## 3. Discussion

Previous reports have shown that regeneration across long (4 cm) ANAs, when compared to short (2 cm) ANAs, is limited by the microenvironment that developed within the graft rather than deficiencies in the ability of neurons to regenerate axons across long distances [12,13,14]. These previous reports focused on the early regenerative processes that impacted these microenvironments. Therefore, the focus of our current study was to further characterize the graft microenvironments of ANAs by considering a longer period of regenerative changes. Through this extended period of consideration, we were able to identify previously undocumented changes to blood vessels and associated changes to inflammatory processes within long versus short ANAs, which could further explain the limited axon regeneration across long versus short ANAs.

Our results were consistent with previous studies showing limited regeneration across 4 cm ANAs [12,13,14]. In this study, we observed that the proximal graft of the short and long ANAs were permissive to axonal regeneration, while the mid-distal graft of the long ANAs had limited regeneration. Similarly, we observed decreased expression of SC markers, concurrent with limited axon regeneration in the mid-distal graft of long ANAs. However, from the 4-week to the 8-week endpoints, we observed an increase in the number of myelinated axons in the mid-distal graft of the long ANAs. This suggests that axons are trying to grow across the microenvironment within long ANAs, even after the 4-week endpoint, although this growth is limited when compared to the short ANAs.

To further explore the differences between the microenvironment of short and long ANAs, we studied the blood vessels within ANAs. Angiogenesis is known to play a critical role in promoting regeneration across nerve grafts, and the disruption of this process leads to delayed and limited regeneration [10,25]. In our study, we found no differences in the density of blood vessels between the short and long ANAs. However, we observed differences in the morphology of the blood vessels within the mid-distal graft of long ANAs. Qualitative analysis of these morphological changes showed decreased blood vessel lumens and increased endothelial cell size. These changes in blood vessel morphology have been reported in different pathologies and are characteristic of process capillary rarefaction [29,30,31]. Previous studies have suggested that this process is driven by the continuous infiltration and activation of immune cells after injury. The activation of these infiltrating immune cells causes the development of a pro-inflammatory environment that is hypothesized to cause the pathological changes observed in blood vessels. This process leads to the formation of a hypoxic environment due to damaged blood vessels, which will then cause the infiltration of immune cells and exacerbate inflammation [29,30,31,32,33,34,35,36]. Failure to resolve this continuous inflammation could lead to fibrosis or tissue destruction and prevent robust tissue regeneration [30,31,37,38,39].

Since inflammation is known to orchestrate nerve regeneration, and the morphological changes observed in the blood vessels of long ANAs suggest the development of a pro-inflammatory environment, we compared the gene expression of inflammatory genes between the short and long ANAs. Comparison of the gene expression of cytokines between ANAs at the 4-week endpoint showed an increase in the macrophage-recruiting gene *Ccl2* in the mid-distal graft of short ANAs. However, by the 8-week endpoint, we observed an increase in the expression of pro-inflammatory genes *Il-6* and *Ifn-γ* and a decrease in the anti-inflammatory gene *Tgf-β* in the mid-distal graft of long ANAs. Similarly, in the mid-distal graft of the long ANAs, the expression of pro-inflammatory genes *Il-6* and *Ccl2* increased from the 4-week to 8-week endpoints while the expression of *Tgf-β* was reduced. In the context of nerve injury, the expression of *Il-6* and *Ccl2* from SC and macrophages in the injured site promotes the recruitment and activation of immune cells that express the pro-inflammatory cytokine *IFN-γ* [24,40,41,42,43]. Exogenous IFN-γ has been shown to polarize macrophages into a pro-inflammatory phenotype and limit axonal regeneration in a conduit model [44,45,46]. Therefore, the increased expression of inflammatory genes within the mid-distal graft of long ANAs could limit axonal growth. On the other hand, the expression of Tgf-β has been suggested to promote SC proliferation, the secretion of neurotrophic factors, and the modulation of immune cells into an anti-inflammatory phenotype [47,48,49,50]. The addition of exogenous Tgf-β after nerve injury also promotes SC reactivation and axonal regeneration in vivo [47]. The decrease in *Tgf-β* expression in the mid-distal graft of long ANAs could disrupt regeneration by limiting SC proliferation or by preventing the modulation of immune cells into an anti-inflammatory phenotype. Interestingly, for short ANAs, we observed an increase in the expression of anti-inflammatory cytokine *Il-10* from the 4-week to the 8-week endpoints. Previous studies have shown that the disruption of Il-10 expression leads to limited axonal regeneration, while the exogenous addition of Il-10 after nerve injury promotes the expression of anti-inflammatory genes in macrophages and robust axonal regeneration [43,51,52]. The increase in *Il-10* expression in short ANAs could limit the formation of a pro-inflammatory environment, promoting a permissive environment for nerve regeneration. The differences in gene expression between the mid-distal graft of short and long ANAs suggest that the microenvironment in long ANAs becomes more pro-inflammatory as time progresses. Interestingly, this coincides with the morphological changes in blood vessels. As a result, inflammation in long ANAs could limit nerve regeneration by damaging newly formed axons and blood vessels, preventing axonal growth. Further experiments revealed no differences in the number of macrophages between grafts at the 8-week endpoint, suggesting that the number of macrophages does not cause differences in gene expression observed between short and long ANAs. Future work will focus on identifying differences in the subpopulations of macrophages between short and long ANAs.

Although we did not observe differences in the density of blood vessels between short and long ANAs, we observed a significant decrease in the expression of *Vegf* in the proximal and mid-distal grafts of long ANAs. Interestingly, *Vegf* expression coincided with an increase in blood vessel density observed from week 4 to week 8 in both grafts. Previous studies have shown threefold and twofold decreases in the expression of endothelial cell marker RECA-1+ in the mid-graft of the long ANAs at 2- and 4-week endpoints, respectively [14]. It is possible that the decreased expression of *Vegf* reduces endothelial cell proliferation in long ANAs. However, angiogenesis is a complex process that is initiated by endothelial cell migration and proliferation, followed by the maturation of the newly formed blood vessels [53,54]. Although the increased expression of RECA1+ in short ANAs at 2 and 4 weeks suggests the presence of more endothelial cells, the measurement of blood vessel density using histology suggests no differences in the formation of mature blood vessels.

A limitation of this study was observing differences in the proportion of functional classes of regenerating fibers in the short versus long ANAs. Human studies have shown higher recovery rates for sensory versus motor modalities across long ANAs [55]. It is hypothesized that sensory recovery is more robust because the small size of sensory fibers allows them to grow efficiently across the basal lamina tubes, and/or there is compensation from adjacent nerves [56,57]. The regeneration of different types of nerve fibers will also depend on the signaling cues present in the microenvironment. For example, the expression of inflammatory proteins can alter the proportion of regenerating sensory and motor nerve fibers [58]. In decellularized nerve grafts, the type of nerve (motor, sensory, or mixed) can also impact the number of regenerating fibers [57]. Although long ANAs have limited regeneration, future work will focus on identifying the functional characteristics and modalities of nerve fibers in proximal and mid-distal grafts.

Recently, commercially available ANAs have been marketed as a new “standard of care” to repair nerve gaps, where these “demonstrate comparable outcomes to autograft in both mixed, motor and sensory nerve gap repair”, in short and long gaps [8]. However, knowing that ANAs and autografts fail with increasing length and diameter is critical to studying the factors associated with failed nerve regeneration across long gaps as we strive to identify a substitute superior to the “gold standard” autograft.

Overall, our studies showed reduced axon regeneration concurrent with morphological changes in the blood vessels and the development of a pro-inflammatory state within long versus short ANAs. Although previous studies have demonstrated that the initial wave of acute inflammation is necessary for angiogenesis and subsequent regeneration, a continuous inflammatory state, or chronic inflammation, can induce blood vessel damage, demyelination, vasculitis-induced nerve ischemia, and the inhibition of axonal growth [28,38,39,59,60,61,62]. Therefore, the development of a pro-inflammatory environment after the 4-week endpoint could play a role in limiting nerve regeneration across long ANAs. Future work will focus on modulating the inflammatory microenvironment within long ANAs to study the effects of inflammation on blood vessel biology and nerve regeneration.

## 4. Materials and Methods

### 4.1. Experimental Design and Animals

Commercially available adult rats (200–250 g, Charles River Laboratories, Wilmington, MA, USA) were utilized for all experiments. Sprague Dawley rats were used as nerve donors to generate ANAs. For the experimental groups receiving ANAs, Lewis rats were used. Surgical procedures and peri-operative care measures were conducted in compliance with the AAALAC-accredited Washington University Institutional Animal Care and Use Committee (IACUC) and the National Institutes of Health guidelines. All animals were housed in a central animal care facility and provided with food (PicoLab rodent diet 20, Purina Mills Nutrition International, St. Louis, MO, USA) and water ad libitum.

The studies included multiple independent sets of animals in order to measure a variety of different outcome metrics (Table S1) to compare short (2 cm) versus long (4 cm) ANAs. For groups, rat sciatic nerve was transected and repaired using either short (2 cm) or long (4 cm) ANAs, as indicated in Table S1. Studies assessed nerve regeneration, the quantity and morphology of blood vessels, and the inflammatory state. The extent of nerve regeneration and the quantity and morphology of blood vessels was assessed at 2, 4 and 8 weeks post surgery using histology and histomorphometry. To assess the inflammatory state, both 4 and 8 weeks were chosen as endpoints to capture changes in the gene expression of pro- and anti-inflammatory genes within ANAs.

**Figure 9:**
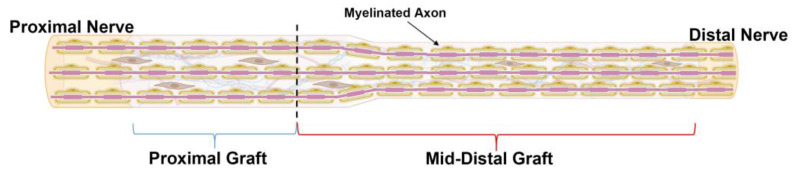
Schematic representation of ANA divided by spatial region of assessment. ANAs were divided into “Proximal graft” (blue bracket) and “Mid-Distal”graft (red bracket) for the assessment of outcome metrics. Black dotted line represents the boundary between these two regions. Created with BioRender.com.

At the endpoints, the harvested tissue was divided into distinct spatial regions. From previous studies, axon regeneration typically starts to dwindle just before the mid-graft of long ANAs [12,13,14]. Therefore, the ANAs were divided into proximal graft (proximal 1/3 of ANA) and mid-distal graft (distal 2/3 of ANA) (Figure 9). This division scheme allowed for the characterization of a region that permits axon growth compared to a region not permissive to axon growth for long ANAs.

### 4.2. Surgical Procedures

To generate ANAs, Sprague Dawley rats were euthanized with an intraperitoneal injection of pentobarbital (150 mg/kg; Fresenius Kabi, Lake Zurich, IL, USA) before performing the subsequent procedures. The sciatic nerve was exposed proximally from the level of the exiting L5-L6 nerve roots at the cruciate ligament to the level of the sciatic nerve trifurcation distally. The length of the harvested grafts yielded 5–6 cm of nerve. The harvested nerves were decellularized (see below) and then trimmed to the appropriate length (2 or 4 cm as necessary immediately before grafting procedures).

For the grafting of the processed nerves, Lewis rats were anesthetized using subcutaneous injections of a ketamine (75 mg/kg; Forth Dodge Animal Health, Fort Dodge, IA, USA) and dexmedetomidine (0.5 mg/kg; Pfizer Animal Health, Exton, PA, USA) cocktail. The right sciatic nerve was exposed by incising the skin and biceps femoris muscle ~1–2 cm below the femur. The exposed sciatic nerve was then transected 4 mm proximal to the distal trifurcation. The grafts were then sutured to repair the gap with 9-0 nylon. The grafted nerves were shaped into multiple loops (“S” shape), and the connective tissue surrounding the grafts was sutured into the caudofemoralis muscle with 11-0 nylon to anchor the graft to the wound area and prevent graft clumping. Following the repair, two-layer muscle and skin closure was performed using a 6-0 vicryl and 4-0 nylon suture, respectively. Atipamezole hydrochloride solution (0.1 mg/kg; Modern Veterinary Therapeutics, Sunrise, FL, USA) was administered for anesthesia reversal, and postoperative pain was managed using Buprenophrine SR (0.05 mg/kg; ZooPharm, Windsor, CO, USA). The animals recovered on a warming pad and were monitored for postoperative complications before returning them to the central animal care facility. Animals were monitored daily for signs of infection, distress, suture dehiscence, and/or lethargy. At the respective endpoints to harvest the grafts, the animals were euthanized, and the procedures were performed just as described above to harvest tissue.

### 4.3. Generation of ANAs

Harvested nerves from the rats were decellularized using a method described previously [13,63]. Briefly, the nerves were repeatedly washed in deionized water, followed by a sodium phosphate buffer containing sulfobetaine-16 (SB-16) and a combination of Triton X-100 and sulfobetaine-10 (SB-10). After processing, the ANAs were washed and stored in a 10 mM phosphate-buffered 50 mM sodium solution at 4 °C until use (within 3 days).

### 4.4. Histology, Histomorphometry and Electron Microscopy (EM) of Nerve

To evaluate nerve regeneration, histology incorporating histomorphometric analyses of the nerve was performed as previously described [64,65]. Collected nerve grafts were fixed via immersion in 3% glutaraldehyde in phosphate buffer and then trimmed into pieces corresponding to the spatial region of the nerve or graft. The samples were then fixed in 1% osmium tetroxide and serially dehydrated using various concentrations of ethanol. The tissues were then embedded in Araldite 506 epoxy resin and cross-sectioned using an ultra-microtome at 1.5 µm sections. The sections were then counterstained with 1% toluidine blue and imaged for analyses at 1000× overall magnification on a Leitz Laborlux S microscope (Leitz Laborlux S; Leica, Buffalo Grove, IL, USA). For analysis, a semi-automated digital image-analysis system linked to morphometry macros developed for peripheral nerve analysis (Clemex Vision Professional, Clemex Technologies, Longueuil, Quebec, Canada) was used. Six-eight random fields per histological section were imaged, resulting in 75–80% of the cross-sectional area being analyzed. Binary histomorphometry analysis of the digitized information based on gray and white scales allowed for measurements of the total fascicular area and total number of myelinated axons in the relevant nerve sections. The quantification of myelinated axons across multiple randomly selected fields per nerve permitted the calculation of the percent of myelinated axons, area covered by myelinated axons (µm^2^), density of myelinated axons (per mm^2^), percent of myelinated axons debris, average size area of myelinated axon, average size area of axon, and average size area of myelin.

Similar to the above, histology with histomorphometry was used for the identification and quantification of the microvasculature and assessment of blood vessel morphology. The quantification of blood vessels permitted the calculation of the density of blood vessels in given fields (per mm^2^). To assess blood vessel morphology, a ratio representing the area of the blood vessel lumen divided by the total area of the blood vessel was used to compare the morphological changes between blood vessels (Figure S10). The numbers for both analysis of nerve regeneration and microvasculature were averaged across the images per animal (n = 1).

Select representative samples obtained at the 8-week endpoint from the proximal and mid-distal grafts of the 2 cm and 4 cm ANAs were imaged to demonstrate microstructural changes in blood vessel morphology using electron microscopy. Specimens already preserved and embedded in epoxy resin were cut into 90 nm cross-sections at random regions along the graft using an LKB III microtome. The sections were then stained with uranyl acetate and lead citrate. Ultramicrographs of the grafts were obtained at 1500–2000× magnification using a JEOL 1200EX electron microscope (JEOL, Peabody, MA, USA).

### 4.5. Immunohistochemistry of Nerve

To additionally assess the regenerative microenvironment, Schwann cells (SCs), axons, blood vessels, and macrophages within the ANAs at the 8-week endpoint were imaged and quantified using immunohistochemistry. Portions of the proximal and mid-distal grafts were immediately placed in 4% paraformaldehyde in phosphate-buffered saline overnight, followed by immersion in 30% sucrose in phosphate-buffered saline (PBS) for 24–48 h. The tissue were then placed in plastic cassettes oriented for cross-sections and frozen in OCT Compound (VWR, Radnor, PA, USA), and then sectioned with a cryostat at 15 µm onto pretreated charged glass slides. The sections were rehydrated in PBS and blocked for 1 h using a solution of 8% normal goat serum, 2% bovine serum albumin, and 0.1% Triton X-100 diluted in PBS. After blocking, a solution containing the primary antibodies diluted in the blocking buffer was applied to the sections and incubated at 4 °C overnight. Primary antibodies were used to stain SCs (S100), axons (β-III tubulin), blood vessels (RECA-1), and macrophages (Iba1) followed by PBS wash and stain for the appropriate fluorochrome-conjugated secondary antibodies for 1 h at room temperature. The concentrations are outlined in Supplementary Table S2. The slides were then washed with PBS, and the sections were mounted with Fluoroshield mounting medium with DAPI (Abcam, Boston, MA, USA) and imaged using a Fluoview F100 confocal microscope and acquisition system (Olympus, Waltham, MA, USA) at 200× (20× water immersion objective) and 600× (60× oil immersion objective) overall magnification. For SC, axon, and blood vessel quantification, the percent area was measured using the ImageJ macro, where positive marker pixels are totaled within a standardized field. For macrophages, the number of Iba1^+^/DAPI cells was quantified within a standardized field. Six sections per graft region and group were analyzed and averaged using ImageJ v1.53t (NIH) to obtain values per animal (n = 1).

### 4.6. Gene Expression Analysis of Nerve

To evaluate the inflammatory microenvironment of ANAs, qRT-PCR was performed with a focus on a broad panel of select genes (Supplementary Table S3). The collected ANAs were divided into proximal and mid-distal graft as before and immediately placed in Trizol (Life Technologies) in liquid nitrogen. The tissue was then thawed and mechanically homogenized, and the RNA was extracted using chloroform and an Rneasy Kit (Qiagen, Valencia, CA, USA) according to the manufacturer’s instructions. The extracted RNA concentration was measured using a NanoDrop 1000 Spectrophotometer (Thermofisher Scientific, Waltham, MA, USA) and adjusted for cDNA preparation. The cDNA was generated with SuperScript II reverse transcriptase (Invitrogen, Carlsbad, CA, USA). To quantify gene expression, real-time PCR was performed using a Step One Plus thermocycler (Applied Biosystems, Foster City, CA, USA) and Taqman Master Mix (Thermofisher Scientific, Waltham, MA, USA) reagents with specific oligonucleotide prime pairs (Supplementary Table S3). The PCR conditions were a hot start at 95 °C for 10 min, followed by 50 °C for 2 min, 95 °C for 15 s and 60 °C for 1 min intervals, repeated for 40 cycles. The gene expression of the selected genes was normalized to an internal control gene (*Actb*). The data were analyzed using Step One Software v3.0.1 (Applied Biosystems, Foster City, CA, USA). Analysis of each spatial region for a group was considered a replicate for each animal (n = 1).

### 4.7. Statistical Analysis

Statistical analysis was performed using GraphPad Prism version 9.5.0 (GraphPad Software, LLC, Boston, MA, USA). Each animal and its nerve represented an ‘n’ value. All data were represented as mean ± standard deviation. Data sets were tested for normality using the Shapiro–Wilk test. Student’s t test was performed for comparison between 2 groups with Gaussian distribution. Welch’s correction was included if the standard deviation between 2 groups was significantly different. For comparison between 2 groups with a non-Gaussian distribution, the Mann–Whitney test was performed. A significance level of *p* < 0.05 was used.

## 5. Conclusions

Limited regeneration across long ANAs is correlated with changes in blood vessel morphology and the development of a pro-inflammatory environment.

## Data Availability

Data sets associated with the paper are available upon request.

## Supplementary Materials

The following supporting information can be downloaded at: https://www.mdpi.com/article/10.3390/ijms25126413/s1.
