# Supplementary material for: Limited Nerve Regeneration across Acellular Nerve Allografts (ANAs) Coincides with Changes in Blood Vessel Morphology and the Development of a Pro-Inflammatory Microenvironment
Source: Int J Mol Sci. 2024 Jun 11;25(12):6413. doi: 10.3390/ijms25126413 (PMC11204013; doi:10.3390/ijms25126413)
Supplement: Supplementary file 1 [file ijms-25-06413-s001.zip › ijms-3035096-supplementary.pdf]

Supplemental Materials

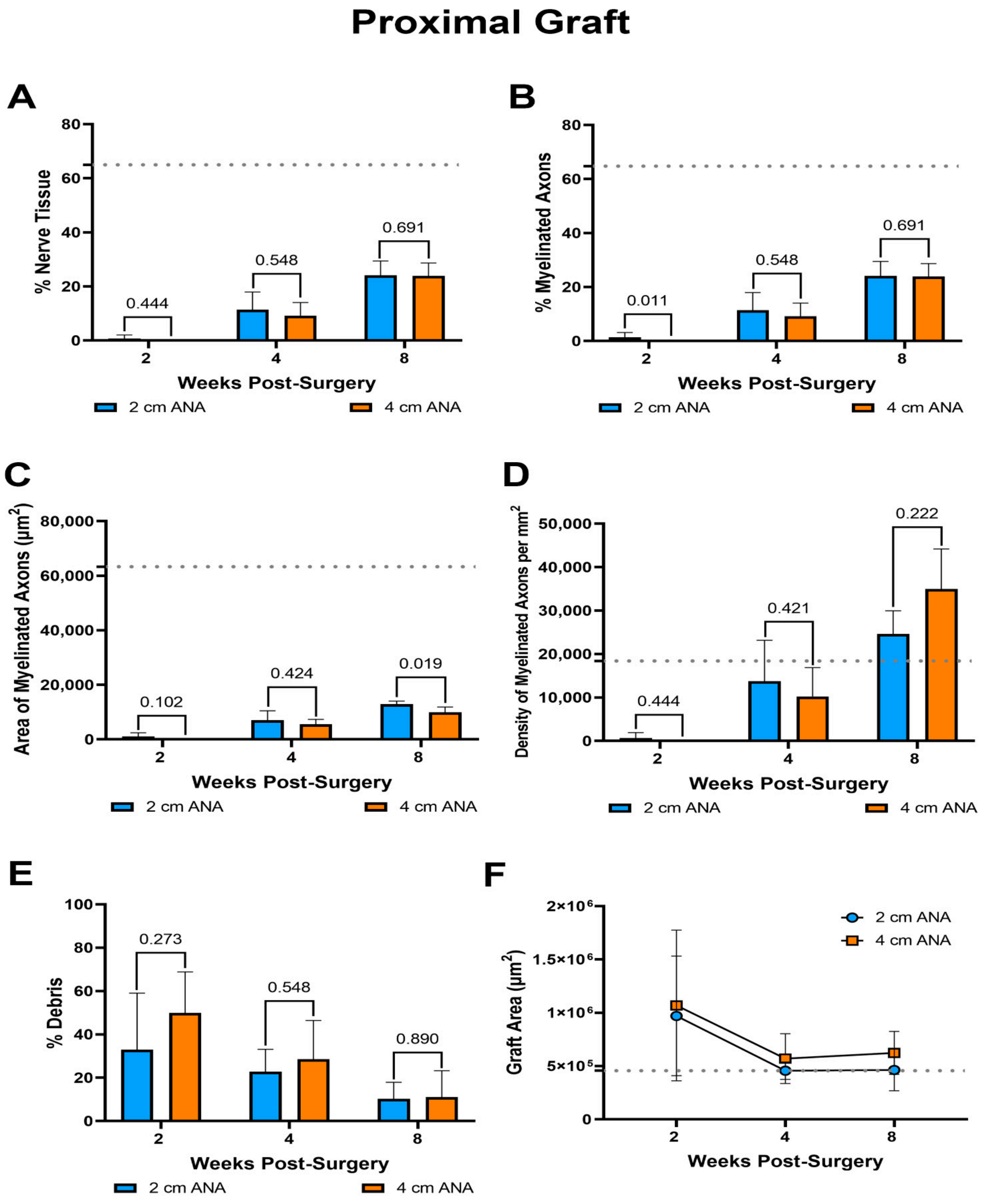

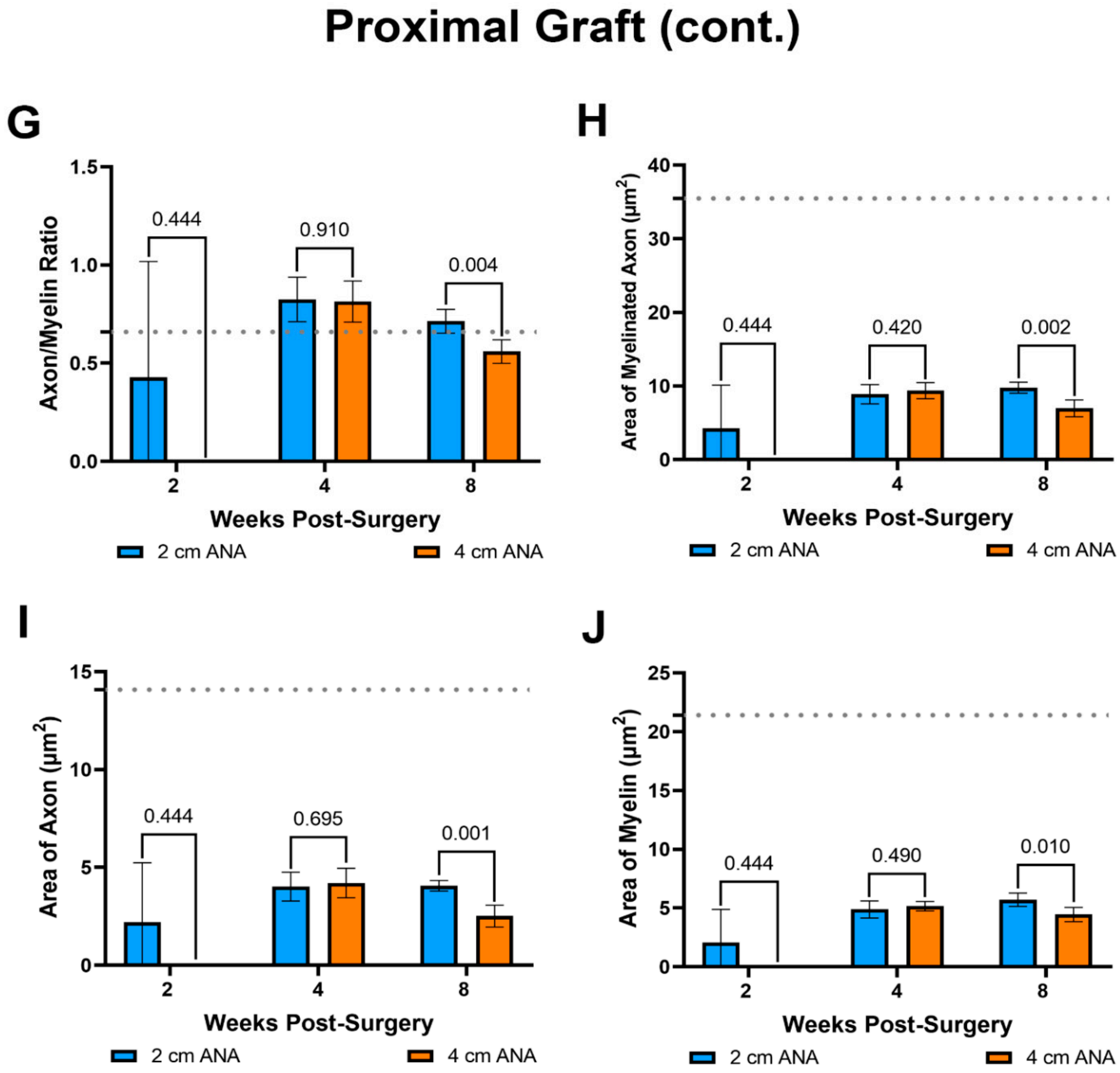

Figure S1. Nerve parameters have minimal differences comparing the proximal graft of the short (2 cm) to long (4 cm) ANAs. Histomorphometric quantification of ANA cross-sections for: A) Percent area of graft with neural tissue; B) Percent area of graft with myelinated axons; C) Area of graft with myelinated axons; D) Density of myelinated axons within graft; E) Percent area of graft with axonal debris; F) Cross sectional area of graft; G) Axon/myelin ratio; H) Area of myelinated axons; I) Area of axons; and J) Area of myelin. Data represented as mean ± SD (n=5/group). P values are represented above each comparison.

Mid-Distal Graft

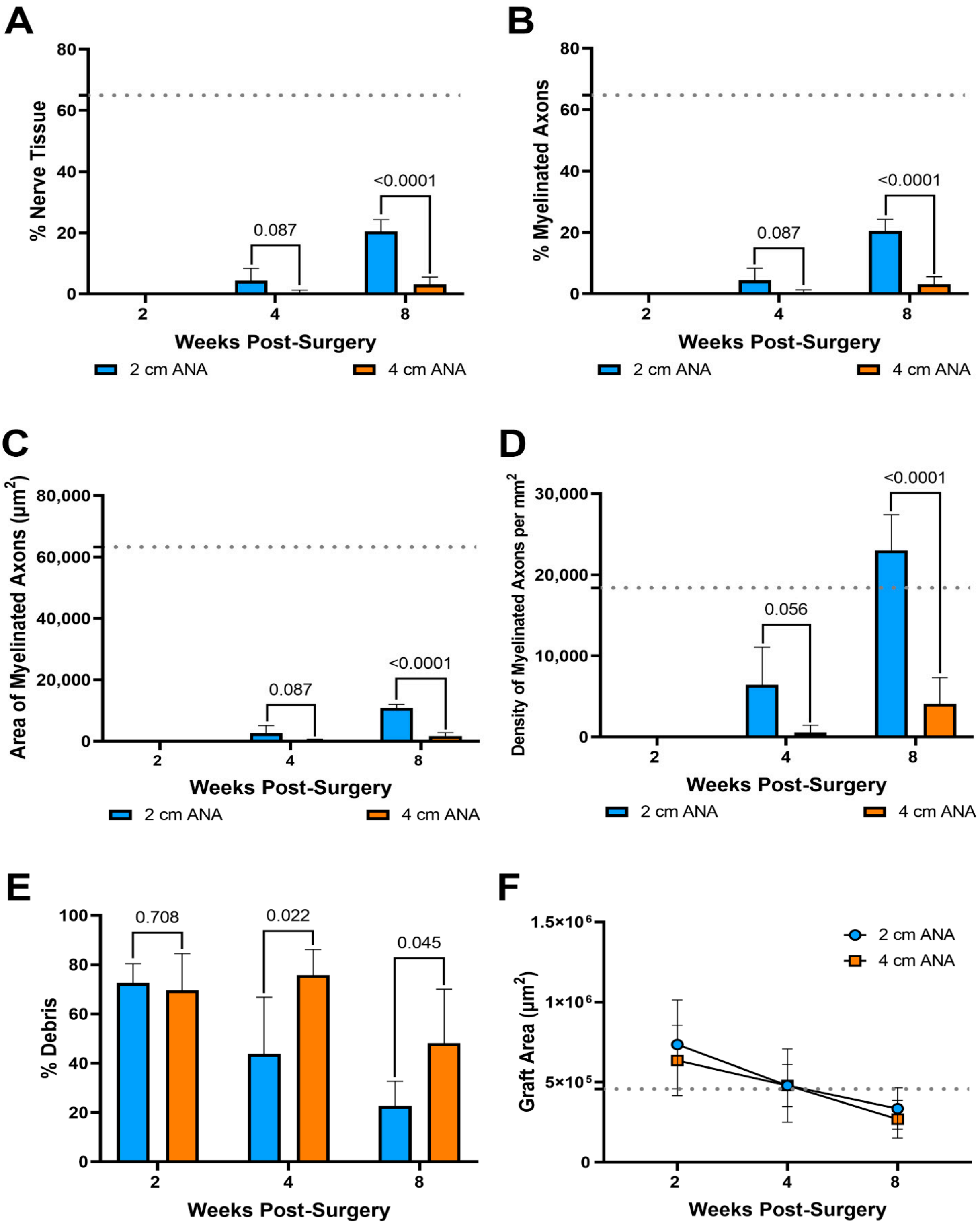

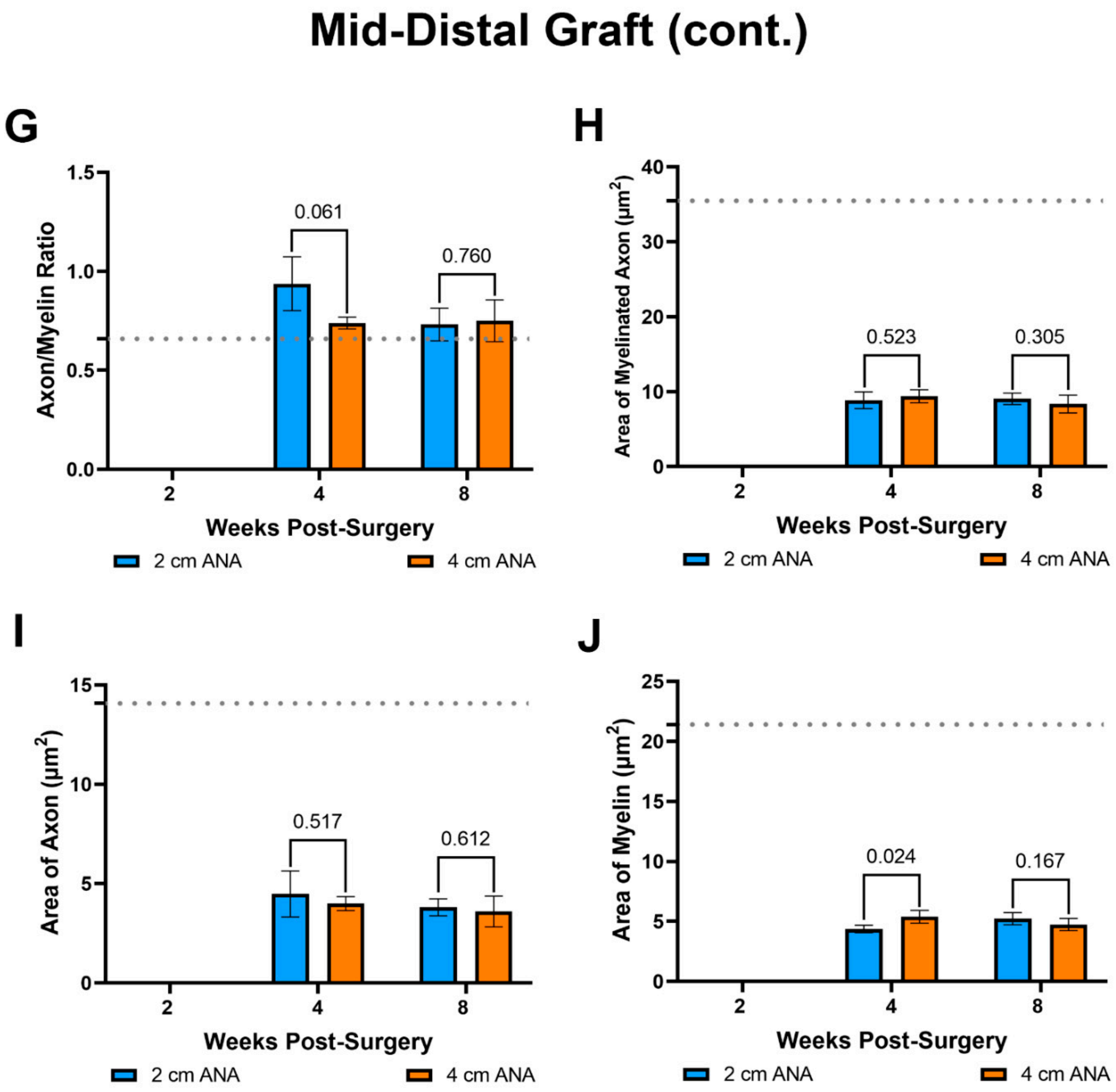

Figure S2. Nerve parameters show limited regeneration within long (4 cm) versus short (2 cm) ANAs at the mid-distal graft. Histomorphometric quantification of ANA cross-sections for: A) Percent area of graft with nerve tissue; B) Percent area of graft with myelinated axons; C) Area of graft with myelinated axons; D) Density of myelinated axons within graft; E) Percent area of graft with axonal debris; F) Cross sectional area of graft; G) Axon/myelin ratio; H) Area of myelinated axons; I) Area of axons; and J) Area of myelin. Data represented as mean ± SD (n=5/group). P values are represented above each comparison.

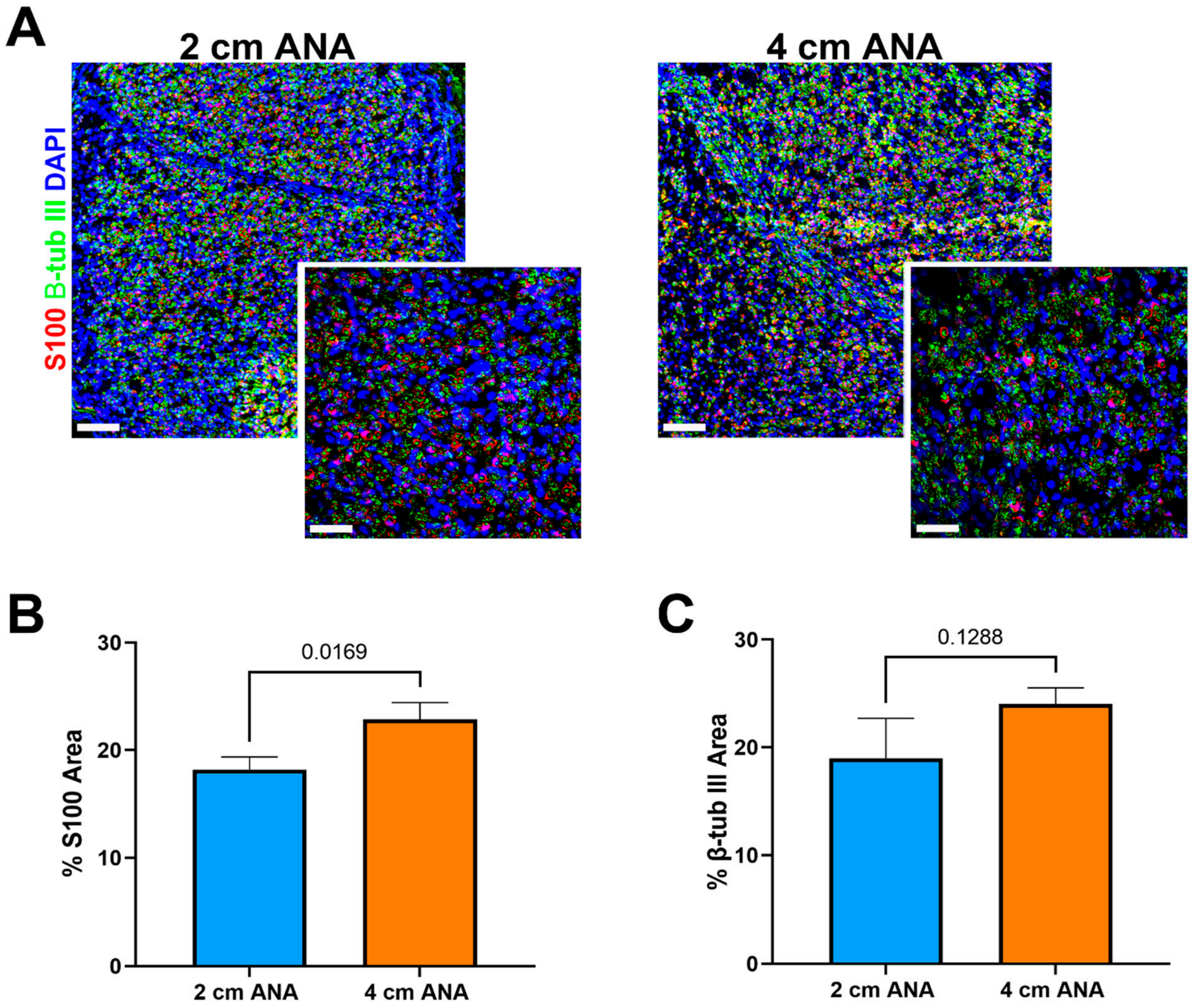

Figure S3. Expression of Schwann cell marker S100 is increased in long (4 cm) versus short (2 cm) ANAs at the proximal graft. A) Immunofluorescence images of graft cross-sections at 8 weeks post-surgery showing SCs (S100; red) and axons (β-III tubulin; green). Quantification of graft cross-sections for percent area stained B) S100 and C) β-III tubulin positive. White scale bar is 30 μm (20x magnification) and 10 μm (60x magnification), respectively. Data represented as mean ± SD (n=3/group). P values are represented above each comparison.

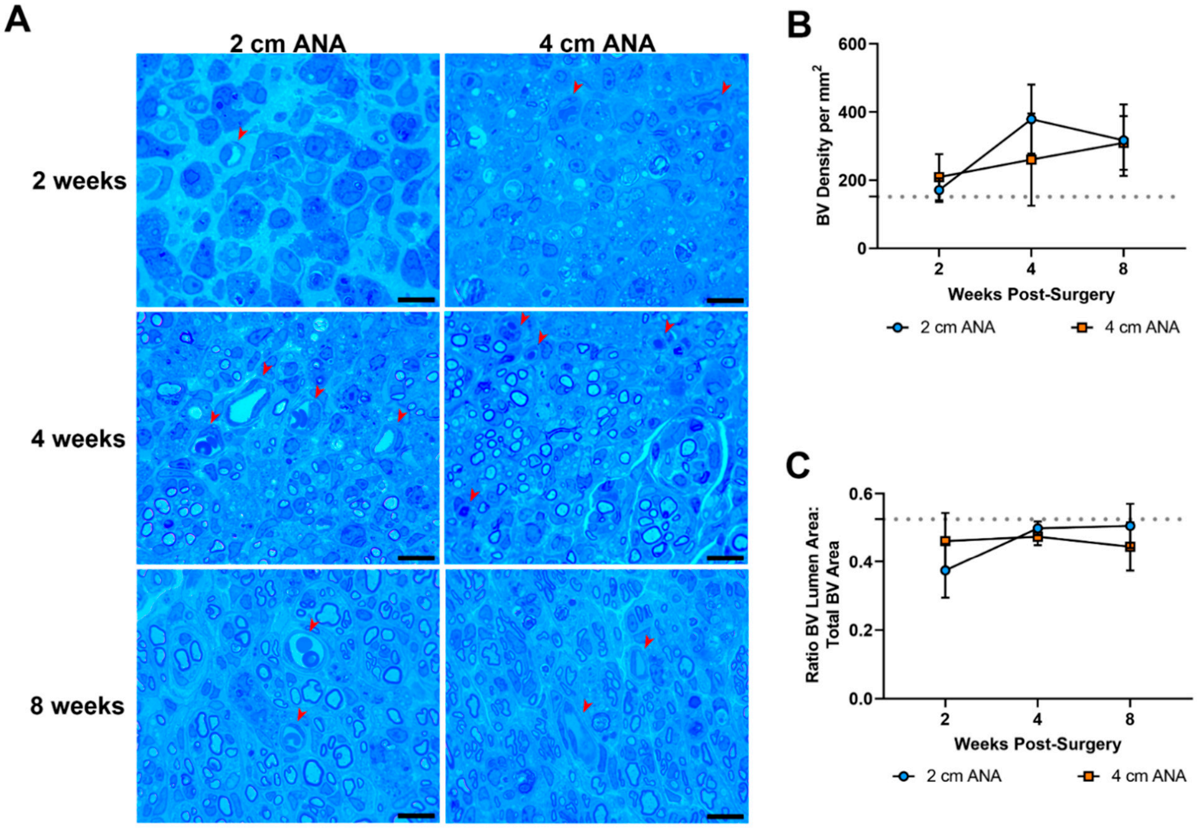

Figure S4. No differences in BV density and morphology comparing short (2 cm) to long (4 cm) ANAs at the proximal graft. A) Representative histological images of graft cross-sections showing BVs (red arrowheads) at 2-, 4- and 8-weeks post-surgery. Black scale bar is 10 μm. Quantification of B) BV density and C) BV morphology. Data represented as mean ± SD (n=5/group). P value is represented next to statistically significant comparisons. Dotted gray line represents the average for uninjured sciatic nerve.

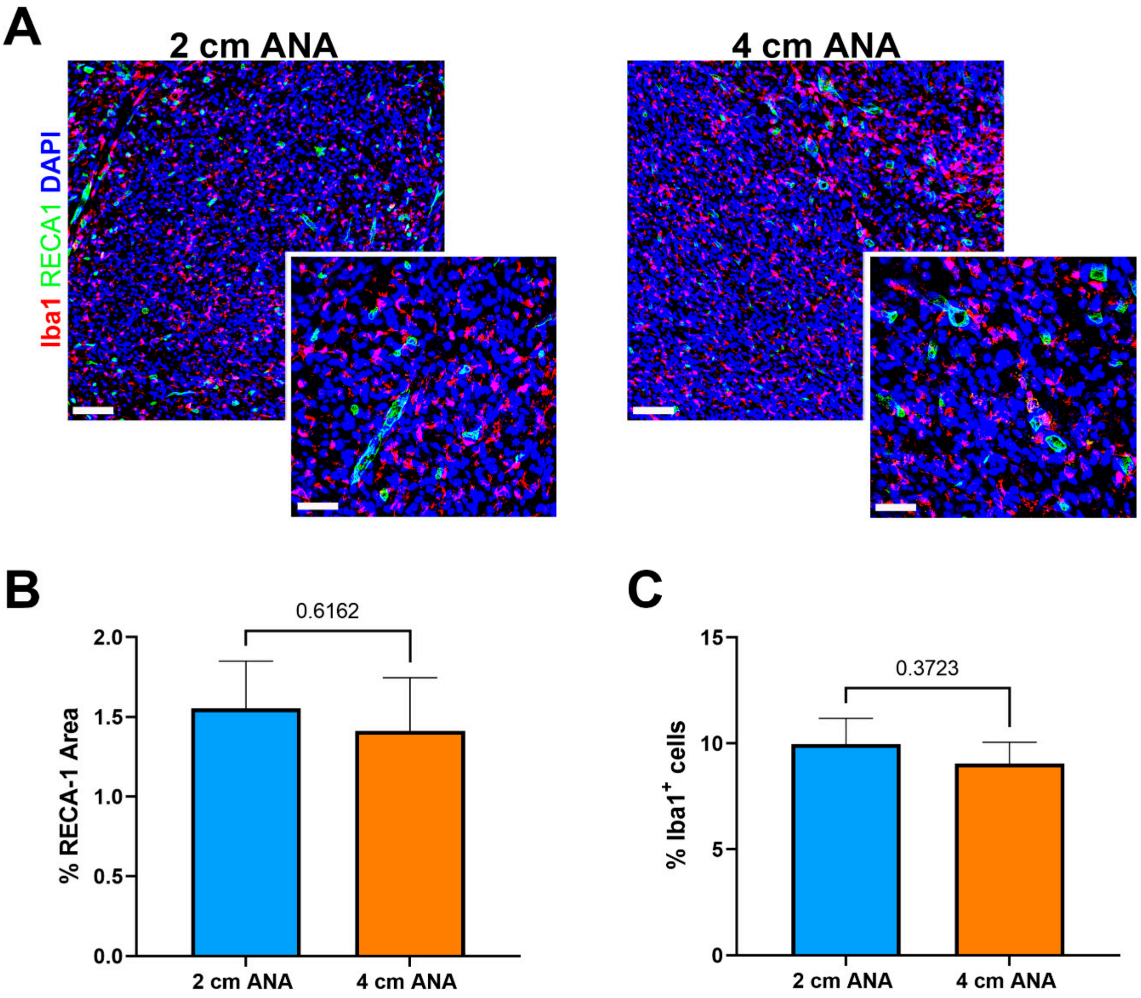

Figure S5. No difference in expression of macrophage marker Iba1 nor endothelial cell marker RECA-1 between the proximal graft of short (2 cm) versus long (4 cm) ANAs. A) Immunofluorescence images of graft cross-sections at 8 weeks post-surgery showing macrophages ($Iba1^+$/DAPI; red/blue) and blood vessels (RECA-1; green). Quantification of graft cross-sections for area stained B) RECA-1 positive and C) $Iba1^+$/DAPI cells. White scale bar is 30 μm (20x magnification) and 10 μm (60x magnification), respectively. Data represented as mean ± SD (n=3/group). P values are represented above each comparison.

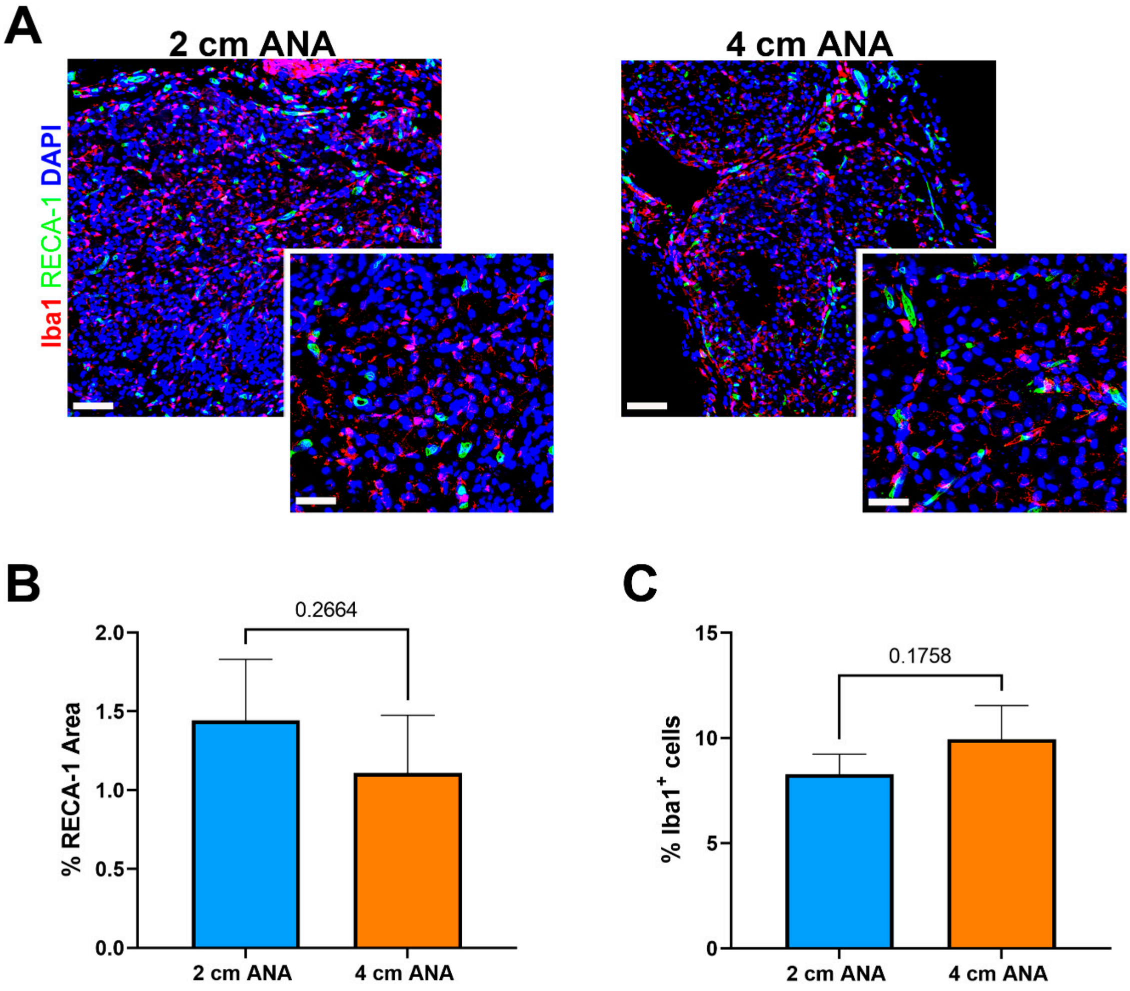

Figure S6. No difference in expression of macrophage marker Iba1 nor endothelial cell marker RECA-1 between the mid-distal graft of short (2 cm) versus long (4 cm) ANAs. A) Immunofluorescence images of graft cross-sections at 8 weeks post-surgery showing macrophages ($Iba1^+$/DAPI; red/blue) and blood vessels (RECA-1; green). Quantification of graft cross-sections for area stained B) RECA-1 positive and C) $Iba1^+$/DAPI cells. White scale bar is 30 μm (20x magnification) and 10 μm (60x magnification), respectively. Data represented as mean ± SD (n=3/group). P values are represented above each comparison.

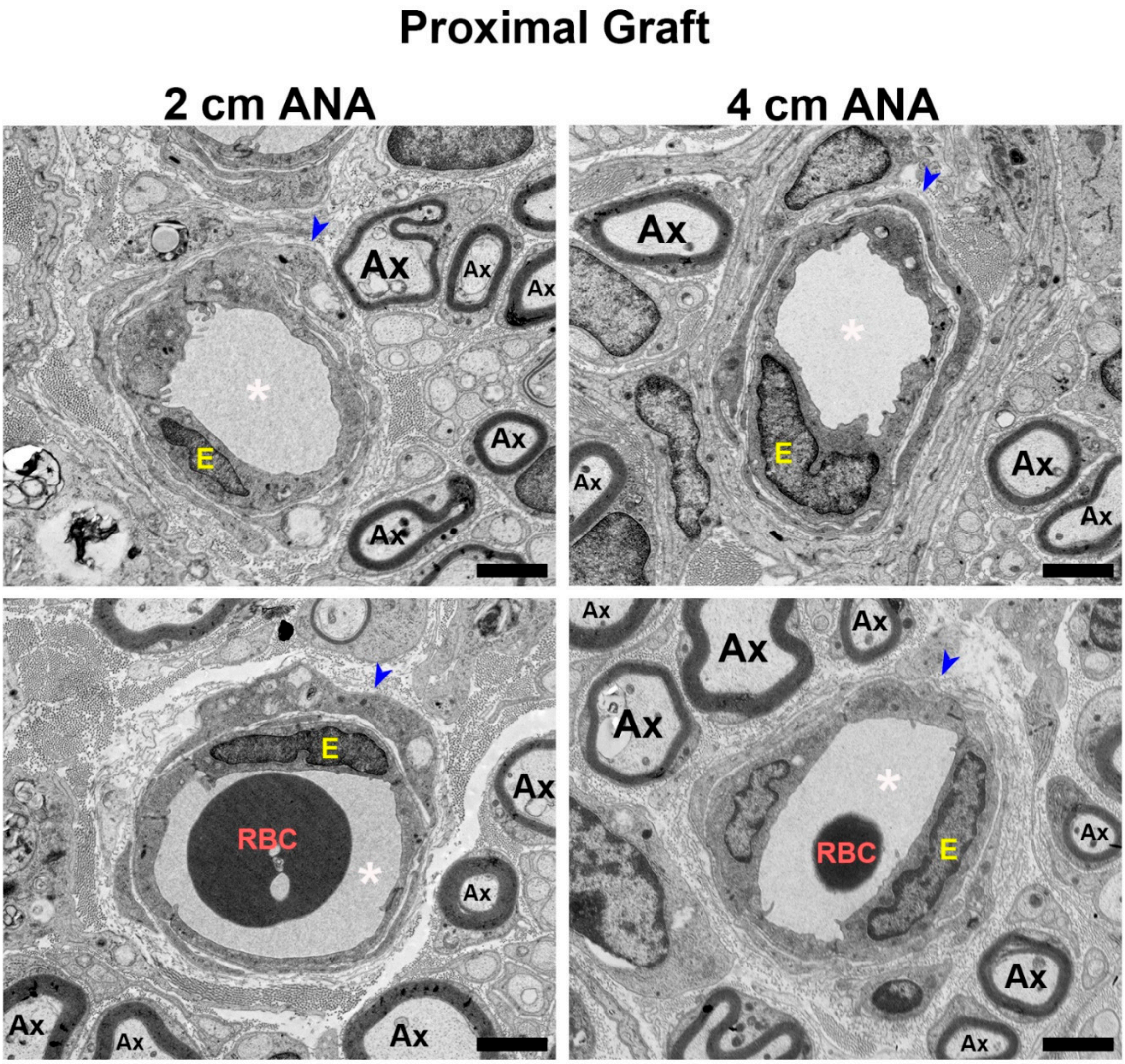

Figure S7. No changes in endothelial cell size and BV lumen comparing short (2 cm) to long (4 cm) ANAs. Representative images from electron microscopy of graft cross-sections show BVs with unobstructed lumen and healthy endothelial cells surrounded by myelinated axons at 8 weeks post-surgery. Blue arrowhead indicate BVs; RBC indicates red blood cells; Ax indicates myelinated axons; white asterisk (*) denotes BV lumen; E indicates endothelial cells. Black scale bar is 2 μm.

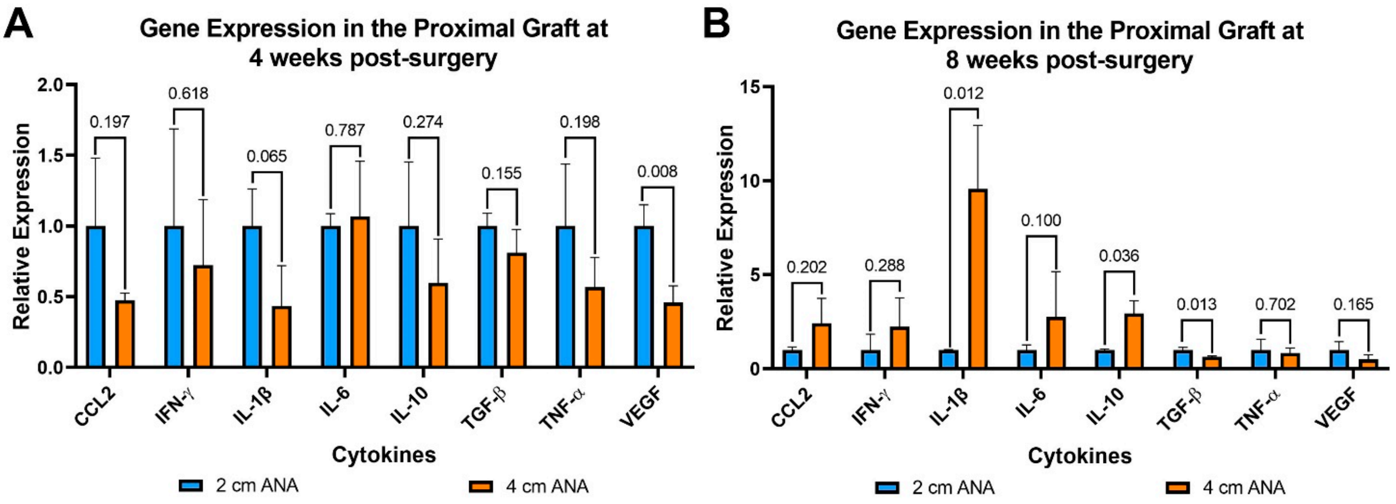

Figure S8. Gene expression of cytokines within long (4 cm) ANAs shows increased inflammation relative to short (2 cm) ANAs. Expression levels are relative to 2 cm ANA measured at A) 4 weeks and B) 8 weeks post-surgery. Data represented as mean ± SD (n=3/group). P values are represented above each comparison.

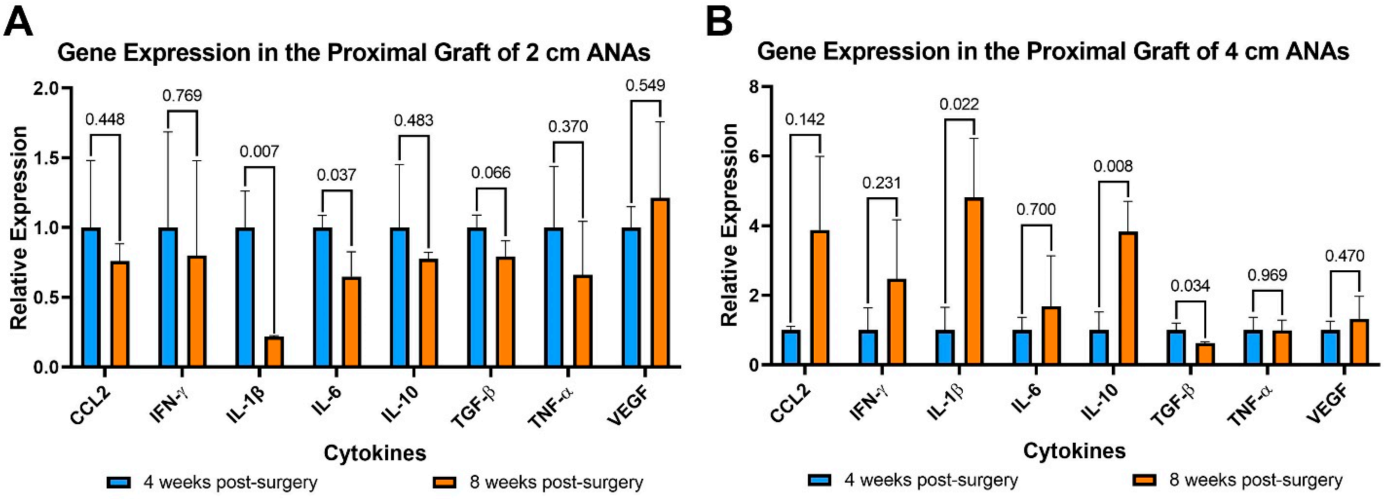

Figure S9. Gene expression of cytokines within long (4 cm) ANAs shows the development of an inflammatory environment over time. Expression levels at 8 week data are relative to 4 week data measured for A) short (2 cm) ANA and B) long (4 cm) ANA. Data represented as mean ± SD (n=3/group). P values are represented above each comparison.

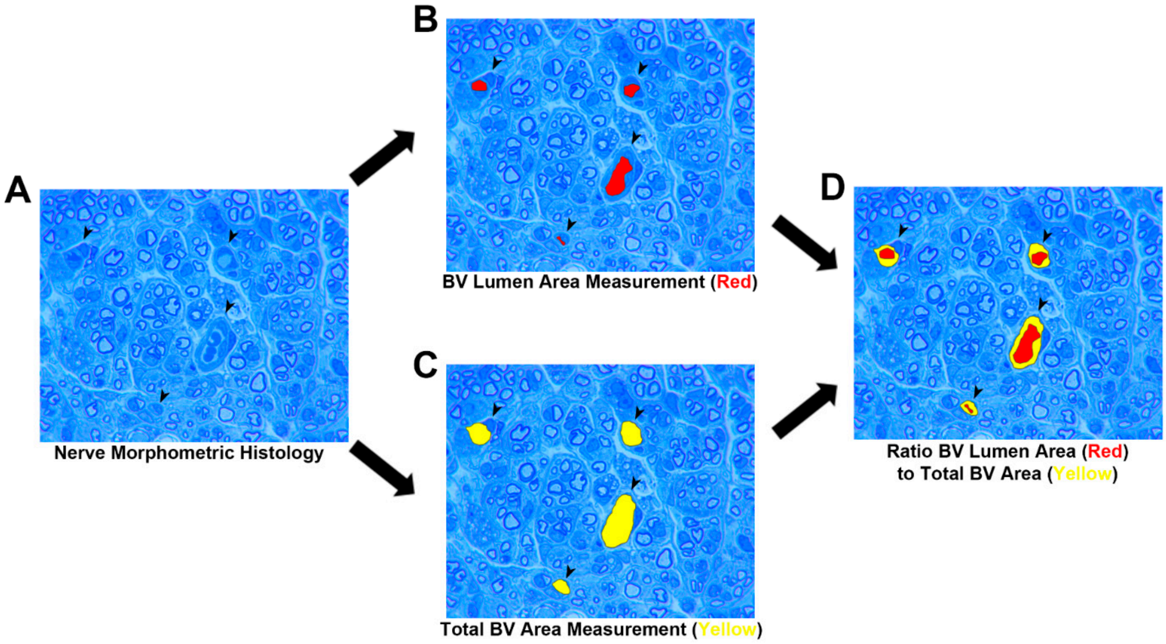

Figure S10. Measurement to assess BV morphology. A) Representative histological image of an ANA cross-section showing BVs (black arrowheads). B) BV lumen area (red) and C) BV total area (yellow) were measured. D) The BV lumen area was divided by the total BV area, providing a ratio between the area of the BV lumen relative to the total area of the BV.

Table S1. Number of animals assigned per experiment.

Animal model	Graft length	Animal number	Endpoint	Analysis
Lewis (M)	2 cm ANA	5	2 weeks	Histology/histomorphometry
		5	4 weeks	Histology/histomorphometry
		5	8 weeks	Histology/histomorphometry, electron microscopy (EM)
	4 cm ANA	5	2 weeks	Histology/histomorphometry
		5	4 weeks	Histology/histomorphometry
		5	8 weeks	Histology/histomorphometry, electron microscopy (EM)
Lewis (M)	2 cm ANA	3	8 weeks	Immunohistochemistry
	4 cm ANA	3	8 weeks	Immunohistochemistry
Lewis (M)	2 cm ANA	3	4 weeks	qRT-PCR of cells contained within ANA
		3	8 weeks	qRT-PCR of cells contained within ANA
	4 cm ANA	3	4 weeks	qRT-PCR of cells contained within ANA
		3	8 weeks	qRT-PCR of cells contained within ANA

Table S2. Antibodies and their dilutions used in this study

	Name of antibody	Supplier	Catalog No.	Dilution
Primary antibodies	S-100	DAKO	GA50461-2	1:5
	β-III tubulin	R&D Systems	MAB1195	1:100
Secondary antibodies	Goat anti mouse Alexa Fluor 488	Thermofisher	A11001	1:500
	Goat anti rabbit Alexa Fluor 555	Thermofisher	A21428	1:500

Table S3. Real time RT-PCR primers used in this study and their Thermofisher Assay ID

Gene name	Assay ID	RefSeq
Ccl2	Rn00580555_m1	NM_031530.1
Ifn-γ	Rn00594078_m1	NM_1388880.2
Il-1β	Rn00580432_m1	NM_031512.2
Il-6	Rn01410330_m1	NM_012589.2
Il-10	Rn99999012_m1	NM_012854.2
Tgf-β	Rn00572010_m1	NM_021578.2
TNF-α	Rn99999017_m1	NM_012675.3
Vegf	Rn01511602_m1	NM_001110333.2
Actb	Rn00667869_m1	NM_031144.3
